# English and American Children

**Published:** 1920-01-24

**Authors:** 


THE PUBLIC WELL-BEING—(*continued*).

### ENGLISH AND AMERICAN CHILDREN.

### Health Comparisons Favour England.

ONE of our leading publicists, who has recently returned from the United States inflamed with the vigour and vitality of American life, has expressed his opinion in the flush of the moment that the Americans have evolved a higher civilisation than that existing elsewhere. It is a sweeping statement, and might be contested on many grounds. But that is too wide a subject for these pages, and is outside their scope. There is, however, an opportune report recently issued by the United States Department of Labour which shows that, at any rate in the health of the children, America still has something to learn from the older civilisation on this side of the Atlantic.

An interesting analysis of the Department's report has appeared in the *Westminster Gazette*. For the purposes of investigation the physical condition of the children was graded in four classes: (1) Excellent; (2) passable; (3) poor; (4) very poor. In making the classification, weight, the general appearance of the child, the condition of the skin, the muscular tone and development, the state of the mucous membrane, the vigour or listlessness which may appear in the child's facial expression, carriage, movements, voice, interest, and attention, all contribute to the decision.

It is estimated that only 173,000 out of the million school children in New York can be placed in Class I.; the "passable" children number 611,000, and the remaining 216,000 are considered to be seriously under-nourished.

It is a striking fact that whilst in England, in spite of the war, the number of seriously under-nourished children has actually decreased, and now amounts to about 10 per cent. of the total, in New York the number of such children is rapidly increasing. In 1914 the number of under-nourished school children was estimated at 5 per cent.; in 1915, 6 per cent.; in 1916, 12 per cent.; and in 1917, 21 per cent. For the United States as a whole, it has been estimated that between 15 and 25 per cent. of school children (3,000,000 to 5,000,000 are under-nourished.

The general conclusion is that poverty, ignorance, and lack of parental control, singly or together, are the factors responsible for such a condition of things as should not exist in a country which possesses the wealth and resources of the States.

A study of English methods of dealing with infantile health has been made, and it is interesting to note that in certain directions American practice is following ours. For example, the beneficial results of school-feeding in England has led some of the largest cities in the States, such as New York, Philadelphia, and Chicago, to start school lunches. These are paid for in many cases, but provision is made for those who are unable to do so. Further, the practice of supplying school dinners has been introduced, and it has been found that an improvement in the health of the children so fed has followed this practice.

### SOME REMEDIAL STEPS.

This must be said of the United States, that it tackles such problems with zeal when once the facts have been revealed. Though not directly arising out of the Labour Department's report, a public health education campaign is in progress with the hope of improving physical standards. It was a matter of some consternation that at America's entry into war one out of every four young recruits examined was found to be unfit for military service. The American girl has been saddled with responsibility because she pays insufficient regard to the laws of health, and the Women's Medical College of Philadelphia has shown initiative in dealing with the matter. A social hygiene board is in existence, and works through the schools and colleges. Sex education is made an important feature. Trained women doctors are engaged, and woman's part in the improvement of the race is emphasised in every particular, including that of dress and food.

